# A resting-state EEG dataset for sleep deprivation

**DOI:** 10.1038/s41597-024-03268-2

**Published:** 2024-04-24

**Authors:** Chuqin Xiang, Xinrui Fan, Duo Bai, Ke Lv, Xu Lei

**Affiliations:** 1 Sleep and NeuroImaging Center, Faculty of Psychology, Southwest University, Chongqing, 400715 China; 2 State Key Laboratory of Space Medicine, China Astronaut Research and Training Center, Beijing, 100094 China

**Keywords:** Cognitive neuroscience, Human behaviour

## Abstract

To investigate the impact of sleep deprivation (SD) on mood, alertness, and resting-state electroencephalogram (EEG), we present an eyes-open resting-state EEG dataset. The dataset comprises EEG recordings and cognitive data from 71 participants undergoing two testing sessions: one involving SD and the other normal sleep. In each session, participants engaged in eyes-open resting-state EEG. The Psychomotor Vigilance Task (PVT) was employed for alertness measurement. Emotional and sleepiness were measured using Positive and Negative Affect Scale (PANAS) and Stanford Sleepiness Scale (SSS). Additionally, to examine the influence of individual sleep quality and traits on SD, Pittsburgh Sleep Quality Index (PSQI) and Buss-Perry Aggression Questionnaire (BPAQ) were utilized. This dataset’s sharing may contribute to open EEG measurements in the field of SD.

## Background & Summary

In contemporary society, insufficient sleep is a prevalent phenomenon with significant implications for both physical and mental health. Research indicates that inadequate sleep duration may impair endothelial function, and disrupt the balance of the autonomic nervous system, thereby increasing the risk of cardiovascular diseases^1^. Insufficient sleep also impacts cognitive functions, including alertness, mood, attention, and memory, as well as various complex cognitive processes such as mental flexibility, planning, and sequencing^2^. Sleep deprivation (SD), as an effective experimental method for studying the effects of insufficient sleep, can aid in a better understanding of the relationship between insufficient sleep and cognitive aspects.

Resting-state electroencephalogram (EEG) is a sensitive brain activity recording for SD. Neurons in the human cortical layer typically process information through electrical signals, allowing their activity to be recorded via EEG^3^. Resting-state EEG captures brain activity in awake individuals without specific cognitive tasks, demonstrating high levels of reliability in terms of split-half and test-retest measures. SD induces significant changes in the frequency bands of resting-state EEG, particularly in the theta (4–7 Hz) and alpha (8–13 Hz) bands. The variability in individual responses to SD is evident in resting-state EEG, and individual sleep sensitivity may be associated with patterns of resting-state EEG changes. In summary, EEG with its high sampling rate is a valuable approach for studying SD.

Our laboratory has published numerous articles combining EEG with SD. For example, Zhang *et al*. conducted a study on the impact of SD on reactive aggression and observed individual variability in the effects of SD on reactive aggression. Their findings indicated that under non-sleep-deprived conditions, gamma power in the prefrontal cortex (PFC) could predict the propensity of individuals to exhibit increased reactive aggression after SD^4^. In addition, Duan *et al*. focused their research on the influence of SD on pain empathy. They revealed that manipulating SD led to a reduction in the subjective pain judgments of pain images, impairing the ability to share others’ experiences of pain without affecting the cognitive processing of empathy for pain^5^. Furthermore, Wang *et al*. investigated the connectivity of the brain’s default mode network under the impact of SD. Their study disclosed adverse effects on the dynamic characteristics of internal connections within the default mode network, particularly a weakened connection between the posterior cingulate cortex and the anterior medial prefrontal cortex, which was closely associated with emotional decline^6^.

The main effect of SD can be manifested through various indicators. The changes in alertness after SD can be assessed through the Psychomotor Vigilance Task (PVT) test^7–9^. Previous studies have shown that participants exhibit noticeable deficits in vigilant attention after just one day of complete SD^10^. Variations in individual characteristics contribute to differential effects of SD on relative attention, as validated in baseline PVT features^11^. SD leads to changes in PVT performance, reflecting response time indicative of errors of commission. Additionally, the Stanford Sleepiness Scale (SSS) and the Karolinska Sleepiness Scale (KSS) serve as subjective indicators for measuring the degree of sleepiness induced by SD. Deterioration in PVT performance and subjective sleepiness may share similar cortical activation patterns, as evidenced by a positive correlation between fast reaction times (Fast RTs) and increased theta activity. This dataset records PVT, SSS and KSS scales in both SD and normal sleep (NS) conditions, serving as indicators of successful SD experimentation and supporting the study of alertness^12^.

There is substantial evidence indicating that SD has a widespread impact on emotional processes. Positive and Negative Affect Schedule (PANAS) is employed to measure individuals’ emotional states at specific moments, showing influences on both positive and negative emotions. Specifically, it manifests as a reduction in positive affect and an increase in negative affect^13^. Research suggests that SD significantly increases depressive and anxiety symptoms while decreasing positive emotional levels in non-clinical adults and adolescents^14^. State Anxiety Inventory (SAI) has demonstrated significant results indicating increased anxiety after SD^15^. Concerning other emotions, tools such as the Buss-Perry Aggression Questionnaire (BPAQ) are utilized to assess aggressive behavior in individuals.

The dataset^16^ shared in this paper comprises data on resting-state EEG under NS and SD conditions, along with behavioral data related to mood and sleepiness from 71 participants. It is distinctive and research-worthy, reflecting the overall impact of SD on all participants and highlighting individual differences. In addition to studying the immediate effects of SD on participants’ states (e.g., EEG collection and state scales such as KSS), we also employ questionnaires to record participants’ traits. This dataset’s sharing may contribute to open EEG measurements in the field of SD.

## Methods

### Overall design

**Fig. 1:**
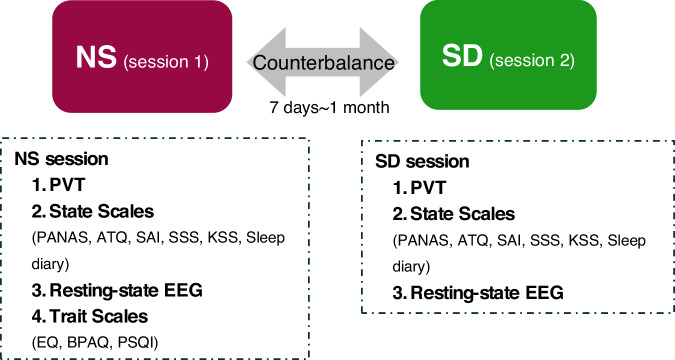
The procedure of resting-state EEG study of sleep deprivation. NS: Normal Sleep, SD: Sleep Deprivation, PVT: Psychomotor Vigilance Task, PANAS: Positive and Negative Affect Scale, ATQ: Automatic Thoughts Questionnaire, SAI: State Anxiety Inventory, SSS: Stanford Sleepiness Scale, KSS: Karolinska Sleepiness Scale, EQ: Empathy Quotient, BPAQ: Buss-Perry Aggression Questionnaire, PSQI: Pittsburgh Sleep Quality Index.

Data collection was conducted from March 2019 to Oct 2021 in Sleep and NeuroImaging Center, Southwest University. The overall procedure is presented in Fig. 1. This dataset^16^ encompasses all EEG data, and PVT data from two experimental sessions: session 1 is NS and session 2 is SD. The overall design is a within-subject design. The two sessions are ideally aligned within a fixed timeframe in the morning or in the afternoon for each subject. Specifically, the number of participants with a difference within the time of day between NS and SD of less than 1.5 hours was 58, accounting for 81.59% of the total. The NS and SD conditions are counterbalanced across participants to eliminate sequence effects. The duration of deprivation was considered with a requirement between 24 and 30 hours. There was a minimum of a 7-day and a maximum of a one-month interval between SD and NS conditions. During the experiment, data collection was conducted in separate rooms, with the room temperature maintained within the normal environmental range (~25 °C) and dim light condition (<50 Lux).

### Participants

A total of 71 participants were involved in our study. The average age of participants is 20 (1.44) years (range: 17–23). The cohort comprises 34 females and 37 males. All participants reported no history of mental illness, anxiety or depressive symptoms, recent cold symptoms, or sleep issues such as insomnia, sudden awakenings, or perceived difficulty in breathing. This study received approval from the Southwest University Ethics Committee (Ethics approval number: H20039), and all procedures were conducted in accordance with the Helsinki Declaration. Participants were provided with detailed explanations of the study’s purpose and procedures, and informed consent was obtained prior to the commencement of the experiment. We obtained consent from all participants for data publication, as well as informed consent from the guardians of underage participants for one participant with age 17.

### Experimental design

The overall design of this experiment is a within-subject design, with NS and SD sessions randomly assigned to achieve counterbalancing across participants. During the EEG procedure, a 5-minute guided instruction for the resting-state with open eyes was employed, and a small group of participants (n = 38) also underwent an additional five minutes of closed-eye state testing. In terms of behavioral assessments, the experiment included three parts: the Psychomotor Vigilance Test (PVT), state scales package, and trait scales package. Prior to the PVT experiment, an instructor provided an explanation of the experiment, followed by participants completing practice trials and the formal experiment.

### Testing procedure

#### Session 1: Normal sleep (NS)

Participants arrived at the laboratory after experiencing normal sleep in their dormitories. Before conducting the NS experiment, participants undergo sleep quality monitoring using sleep diaries or actigraphs to ensure normal sleep prior to the experiment, thus avoiding potential impacts on the experimental results. Participants were assisted by the experimenter in hair washing and the application of electrode gel. Subsequently, participants engaged in PVT testing, along with assessments for state scales covering mood and sleepiness, including PANAS, Automatic Thoughts Questionnaire (ATQ), State Anxiety Inventory (SAI), SSS, KSS, and the Reduced version of Sleep Diary (Sleep Diary). Upon completion of the scales, EEG data collection was conducted. During the resting-state EEG recording, participants were instructed to fixate on a point for five minutes (eyes open), followed by another five minutes with eyes closed (partially). They were required to remain still, quiet, and relaxed, minimizing eye blinking. Each experimental phase utilized 61 Ag/AgCl active electrodes mounted in an elastic cap, arranged according to the extended 10–20 international electrode placement system (Brain Products GmbH, Steingrabenstr, Germany). FCz served as the online reference electrode. The sampling rate was 500 Hz, and electrode impedance was maintained below 5 kΩ after careful preparation. Finally, participants filled out some trait scales, including Empathy Quotient (EQ), BPAQ, and Pittsburgh Sleep Quality Index (PSQI).

#### Session 2: Sleep deprivation (SD)

In this session, participants arrived at the laboratory at 21:00 on the evening before the experiment. To ensure that participants remained awake throughout the entire experiment, they were continuously monitored with two experimenters during SD period until the end of the experiment on the following day. They were also monitored by an actigraphy wrist-watch (wGT3X-BT). They were prohibited from consuming beverages or foods containing caffeine, tea, or alcohol. Additionally, lying down, sleeping, or engaging in strenuous physical activity was not allowed. After SD, there was no recovery sleep period, and participants immediately underwent the experiment. Then participants took part in the experiment with the same procedure as in NS condition. As NS and SD were counterbalanced between participants, the two sessions were ideally aligned within the same time of day for each subject.

### Released behavioral tests

For each participant, in addition to EEG data, we collected a series of behavioral data. These behavioral data include general demographic information, such as gender and age, as well as state and trait scales. Table 1 summarizes the published information on behavioral variables.

**Table 1:** Released behavioral data.

Name	Test Conditions	Key Variables	Reference	Total Score Range
**State scale package**				
Positive and Negative Affect Scale (PANAS)	NS and SD	Positive and negative affect scores	Watson *et al*. 1998	10–50
Automatic Thoughts Questionnaire (ATQ)	NS and SD	Total score	Hollon and Kendall, 1980	30–150
State anxiety inventory (SAI)	NS and SD	Total score	Spielberger *et al*. 1970	20–80
Stanford Sleepiness Scale (SSS)	NS and SD	Total score	Hoddes *et al*. 1972	1–7
Karolinska Sleepiness Scale (KSS)	NS and SD	Total score	Akerstedt and Gillberg,1990	1–9
Reduced version of Sleep Diary (Sleep Diary)	NS	Factor scores	van Hees *et al*. 2015	1–10
**Trait scale package**				
Empathy Quotient (EQ)	NS	Total score	Baron-Cohen and Wheelwright^17^	0–80
Buss-Perry Aggression Questionnaire (BPAQ)	NS	Total score	Lü, L. *et al*. 2013	22–110
Pittsburgh Sleep Quality Index (PSQI)	NS	Total score and Fctor scores	Buysse *et al*. 1989	0–21

### Released EEG recordings

The final shared resting-state EEG data comprises 71 participants with eyes open and 38 participants with eyes closed^16^. The recording duration was standardized to 5 minutes. Notably, no preprocessing was applied to any of the uploaded data^16^.

## Data Records

**Fig. 2:**
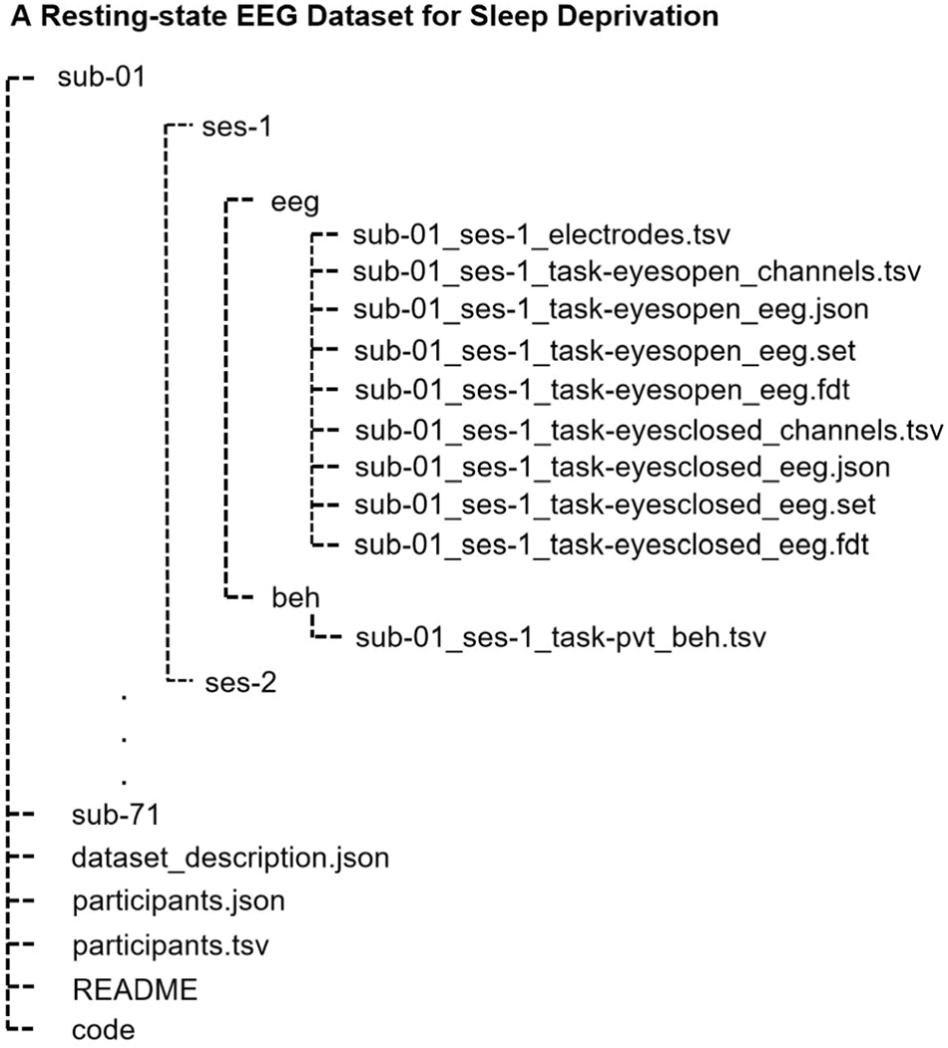
The structure of the dataset in BIDS format.

The dataset^16^ (10.18112/openneuro.ds004902.v1.0.4) is named “A Resting-state EEG Dataset for Sleep Deprivation” and all files are in BIDS format15. The main folder of the dataset contains 71 subfolders, each corresponding to a participant, and four additional files. Each participant’s folder includes two subfolders for two sessions, i.e., NS and SD, encompassing EEG and PVT behavioral data recordings. The EEG consists of open-eye and partially closed-eye EEG data, electrodes, etc. (refer to Fig. 2). The four additional files are: (i) “data-description.json,” providing information on dataset description and registration details, including location and time; (ii) “participants.tsv,” containing participant information such as gender, age, and behavioral data from the mentioned questionnaires; (iii) “participants.json,” describing all columns presented in the “participants.tsv” file; and (iv) “README,” offering general information about the dataset, including contact details.

## Technical Validation

### PVT

**Fig. 3:**
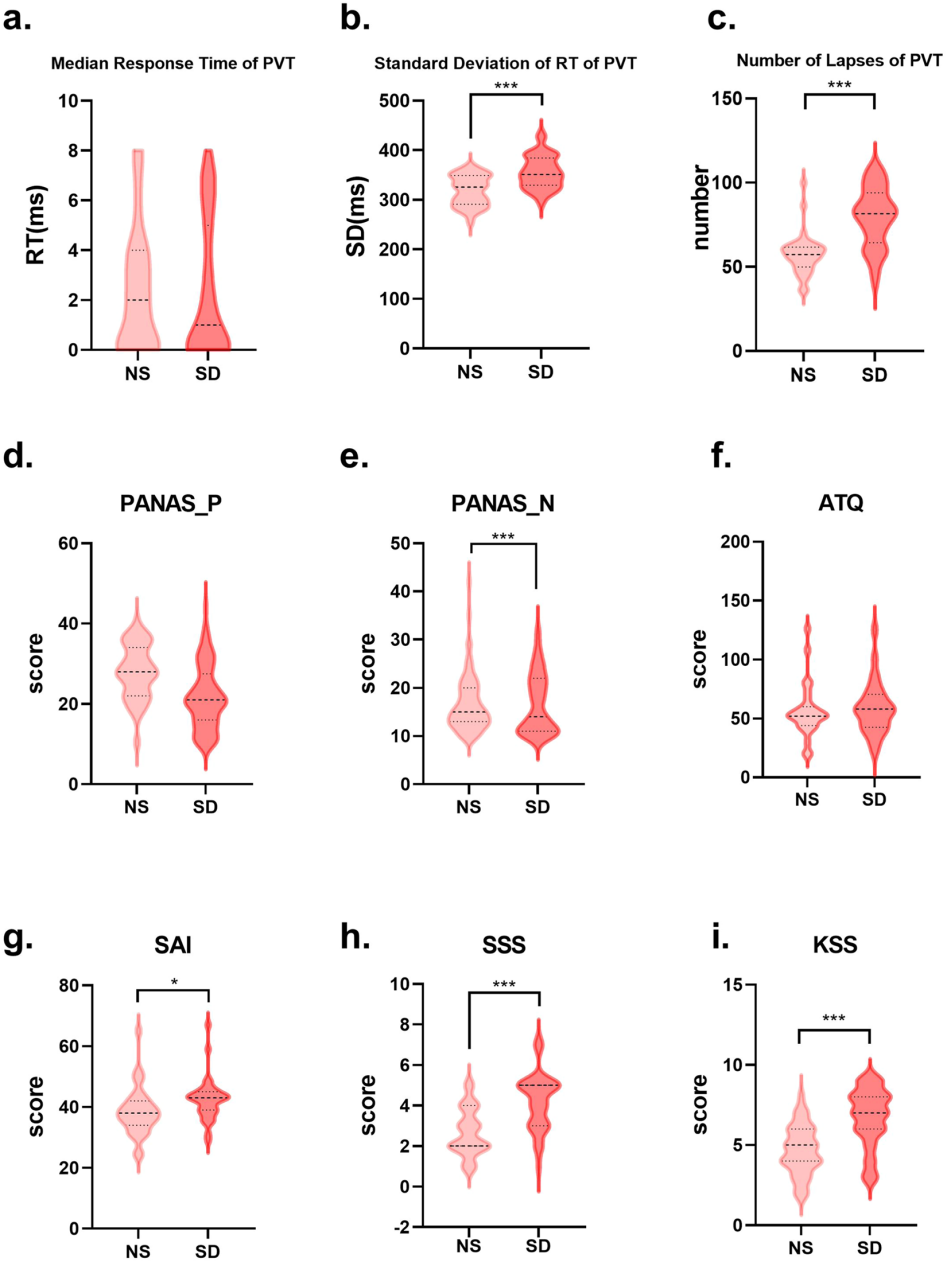
Distribution of PVT, PANAS_P, PANAS_N, ATQ, SAI, SSS, and KSS between two sessions. Median response time, standard deviation of RT and number of lapses are 3 indicators of PVT. ***p < 0.001, *p < 0.05. Abbreviations: PVT, psychomotor vigilance task; PANAS_P, positive affect of Positive and Negative Affect Scale; PANAS_N, negative affect of Positive and Negative Affect Scale; ATQ, Automatic Thoughts Questionnaire; SAI, State anxiety inventory; SSS, Stanford Sleepiness Scale; KSS, Karolinska Sleepiness Scale; NS, normal sleep; SD, sleep deprivation.

To assess changes in participants’ alertness between NS and SD, two PVT sessions were conducted. Based on previous studies, we chose and computed three basic response time indicators (median response time (RT), standard deviation of RT, and number of lapses (RT > 500 ms) (see Fig. 3a–c)^9^. The results indicated significant differences in standard deviation of RT (t(29) = −7.31, *p < *0.001) and number of lapses (t(29) = −4.72, *p < *0.001) between the two sessions. Median reaction time, however, showed no significant change (t(29) = 0.04, *p = *0.97).

### State Scales

To assess changes in positive and negative emotions between NS and SD, the differences in PANAS scores were compared for participants between the two sessions. The results indicated a significant change in participants’ negative emotions (t(67) = −7.89, *p < *0.001). However, there was no significant difference in positive emotions (t(67) = −1.32, *p = *0.19).

To assess differences in participants’ depressive mood, the differences in ATQ scores were compared between the two sessions. The results showed no significant change in participants’ depressive mood (t(24) = −1.47, *p* = 0.15).

To assess differences in participants’ anxiety levels, the differences in ATQ scores were compared between the two sessions. The results indicated a significant change in participants’ anxiety levels (t(30) = −2.35, *p < *0.05).

To assess differences in participants’ sleepiness, the differences in SSS and KSS scores were compared between the two sessions. The results showed a significant change in participants’ sleepiness levels, with SSS at t(34) = −4.73, *p < *0.001, and KSS at t(32) = −5.72, *p < *0.001. Both KSS and SSS serve as subjective indicators of sleepiness.

### Trait scales

Based on the scores from the PSQI (Global Score < 7), it can be concluded that the participant has good sleep habits and quality, and does not use any sleep aid medications. In addition, we also utilized the EQ and BPAQ scales to assess the participant’s empathy and aggression levels. In terms of empathy, the participant is generally at an average level (33–52)^17^. According to the BPAQ, the participant did not exhibit high aggression (<65)^18^. The descriptive statistics of trait scales are presented in Table 2.

**Table 2:** The descriptive statistics of trait scales.

Scales	Mean(SD)	Range
EQ	34.00 (8.54)	17–58
BPAQ	57.30 (10.90)	32–80
PSQI_GlobalScore	5.00 (2.46)	0–12
PSQI_Item1	1.01 (0.83)	0–3
PSQI_Item2	1.00 (0.92)	0–3
PSQI_Item3	0.46 (0.61)	0–2
PSQI_Item4	0.30 (0.78)	0–3
PSQI_Item5	0.78 (0.49)	0–2
PSQI_Item6	0.00 (n.a.)	0
PSQI_Item7	1.43 (0.84)	0–3

n(EQ) = 56, n(BPAQ) = 55, n(PSQI) = 66, EQ: Empathy Quotient, BPAQ: Buss-Perry Aggression Questionnaire, PSQI: Pittsburgh Sleep Quality Index.

### EEG power spectrum

Preprocessing of the eye-open EEG was completed with MATLAB R2021b (The MathWorks) and EEGLAB (version 2021, http://sccn.ucsd.edu/). The general processing pipeline was used. The first step involved bandpass filtering the raw EEG from 0.2 to 45 Hz. After that, a visual examination was carried out, and the 5-minute EEG data was segmentedto 75 epochs with 4 seconds. Epochs containing notable artifacts were eliminated, with an average artifact epoch range of 1.46 (2.02) across all participants. The mean number of bad electrodes was 2.39%, and we interpolated its signal with its surrounding electrodes. Independent Component Analysis (ICA) was employed to remove stereotyped muscle and ocular artifacts; more information is available in this article (Delorme and Makeig, 2004). The signal was finally re-referenced to the average.

**Fig. 4:**
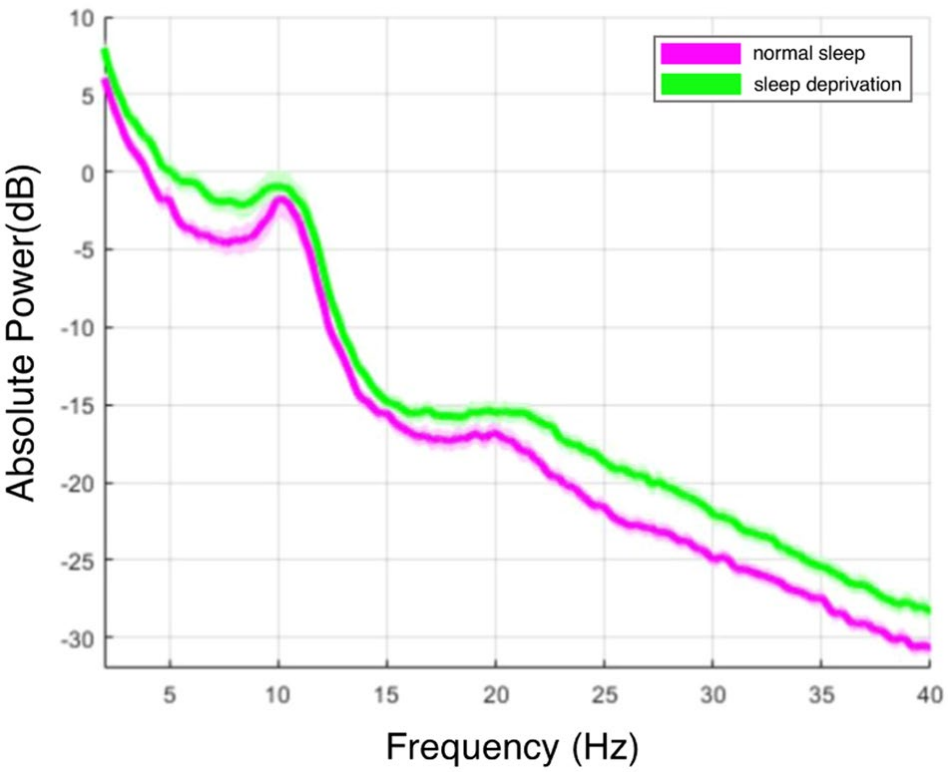
EEG power spectrum during normal sleep (pink) and sleep deprivation (green).

For the preprocessed EEG dataset, we further computed the spectrum for Cz using the Welch method. The absolute power for each electrode was logarithmically transformed to calculate the power spectrum (1 dB = 10 × log(μV²)). The mean and standard deviation for all participants were calculated under the two sessions. Figure 4 illustrates the spectral activity of EEG in both conditions. It is evident that the absolute power during SD is generally higher than NS. The maximum power of EEG in both conditions is concentrated in the low-frequency range (around 0.2 to 13 Hz) with a peak around 10 Hz.

**Fig. 5:**
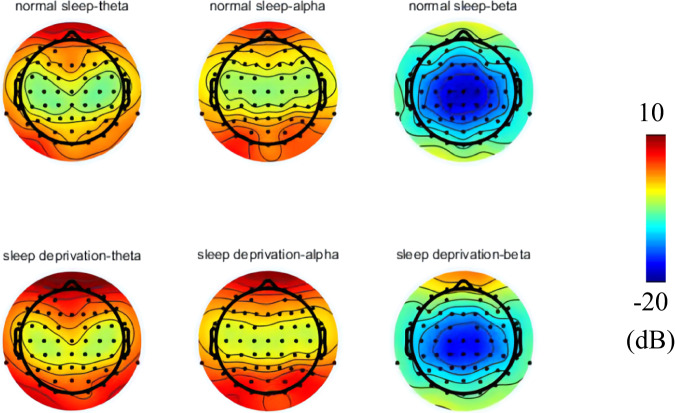
Topography of resting-state EEG in theta, alpha and beta for normal sleep (1^st^ row) and sleep deprivation (2^nd^ row) conditions.

The topographical maps of theta (4–8 Hz), alpha (8–13 Hz), and beta (13–30 Hz) for SD and NS are shown in Fig. 5. In both sessions, the high-power areas of alpha and theta are predominantly distributed around the frontal and occipital lobes. In contrast, beta exhibits a low-energy state primarily in the parietal lobe for both conditions.

## Data Availability

The code used for conducting spectral analysis, and generating topographic maps of the EEG data was uploaded to the *OpenNeuro* dataset along with other data^16^.
